# Hybrid predictor for ground-motion intensity with machine learning and conventional ground motion prediction equation

**DOI:** 10.1038/s41598-020-68630-x

**Published:** 2020-07-27

**Authors:** Hisahiko Kubo, Takashi Kunugi, Wataru Suzuki, Shingo Suzuki, Shin Aoi

**Affiliations:** National Research Institute for Earth Science and Disaster Resilience, 3-1, Tennodai, Tsukuba, Ibaraki 305-0006 Japan

**Keywords:** Natural hazards, Geophysics, Seismology, Computational science, Computer science

## Abstract

The use of strongly biased data generally leads to large distortions in a trained machine learning model. We face this problem when constructing a predictor for earthquake-generated ground-motion intensity with machine learning. The machine learning predictor constructed in this study has an underestimation problem for strong motions, although the data fit on relatively weak ground motions is good. This underestimation problem is caused by the strong bias in available ground-motion records; there are few records of strong motions in the dataset. Therefore, we propose a hybrid approach of machine learning and conventional ground-motion prediction equation. This study demonstrates that this hybrid approach machine learning technology and physical model reduces the underestimation of strong motions and leads to better prediction than either of the individual approaches applied alone.

## Introduction

The rapid progress of machine learning (ML) technology has expanded opportunities for using ML in numerous fields, including geoscience^1,2^. The performance of ML algorithms relies strongly on the quality of the training data. Therefore, strongly biased data can lead to large distortions in a trained ML model. For example, automated facial analysis algorithms produced by major tech companies have gender and skin-type biases mainly because of the use of biased data^3,4^. Data bias is a troublesome and unavoidable problem in ML and is also present in geoscience. For example, accumulated records of earthquake-generated ground motions are highly imbalanced. Although many seismic waves have been recorded since the beginning of modern seismic observations, there are few records of “strong” ground motions, which sometimes cause human casualties and building damage. Because ground-motion data are used in various geoscience fields, the use of biased ground-motion data can negatively affect the performance of ML algorithms used in geoscience. This study focuses on predicting the intensity of ground motions caused by earthquakes (e.g., the seismic intensity or peak ground acceleration) using ML. We investigate how data bias affects ML-based predictors and what we can do to reduce this effect.

When an earthquake occurs, a part of the energy stored in the Earth is released in the form of seismic waves that originate from the source fault, and the seismic waves pass through the earth. When they reach the Earth’s surface, the ground shakes. The intensity of earthquake-generated ground motions depends on complex interactions between the earthquake source, observation site, and path between the source and the site^5^. Because the dependence trend can be modeled using an empirical approach, regression equations that model observed ground-motion amplitudes with the earthquake magnitude, source-site distance, and other factors have been developed from ground-motion records using physical knowledge of the generation mechanism of ground motions^5–11^. These equations, called ground motion prediction equations (GMPEs), are useful for seismic hazard and risk assessments^6^ and earthquake early warning^12^.

Recent studies have applied ML technology to the field of GMPEs^13–16^. The advantages are that (1) nonparametric ML methods can learn functions of the ground-motion models directly from data without the assumption of regression equations and (2) new data or explanatory variables that have not been used in conventional GMPEs can be adopted because of the high flexibility of ML. The introduction of ML is expected to lead to a more useful predictor for ground-motion intensity. However, biases in ground-motion data hinder the potential of ML. In this study, we aim to tackle the problem of data bias directly.

First, we construct a predictor for ground-motion intensity using a nonparametric ML method. However, this predictor has an underestimation problem for strong motions because of the bias in available ground-motion records. To address this problem, we propose a hybrid approach using an ML model augmented by a conventional GMPE.

### Machine learning predictor

We construct an ML predictor with the target ground-motion intensity of peak ground acceleration (PGA). We adopt five explanatory variables: The epicentral distance *D* (horizontal distance between the earthquake and an observation station), moment magnitude *M*_w_, event depth *H*, top depth to the layer whose S-wave velocity is 1,400 m/s at the site (*D*_1400_), and average S-wave velocity up to a 30 m depth at the site (*Vs30*) following Morikawa and Fujiwara^8^. The *D*_1400_ and *Vs30* information is used to represent the site amplification by deep sedimentary layers and shallow soft soils, respectively.

**Figure 1:**
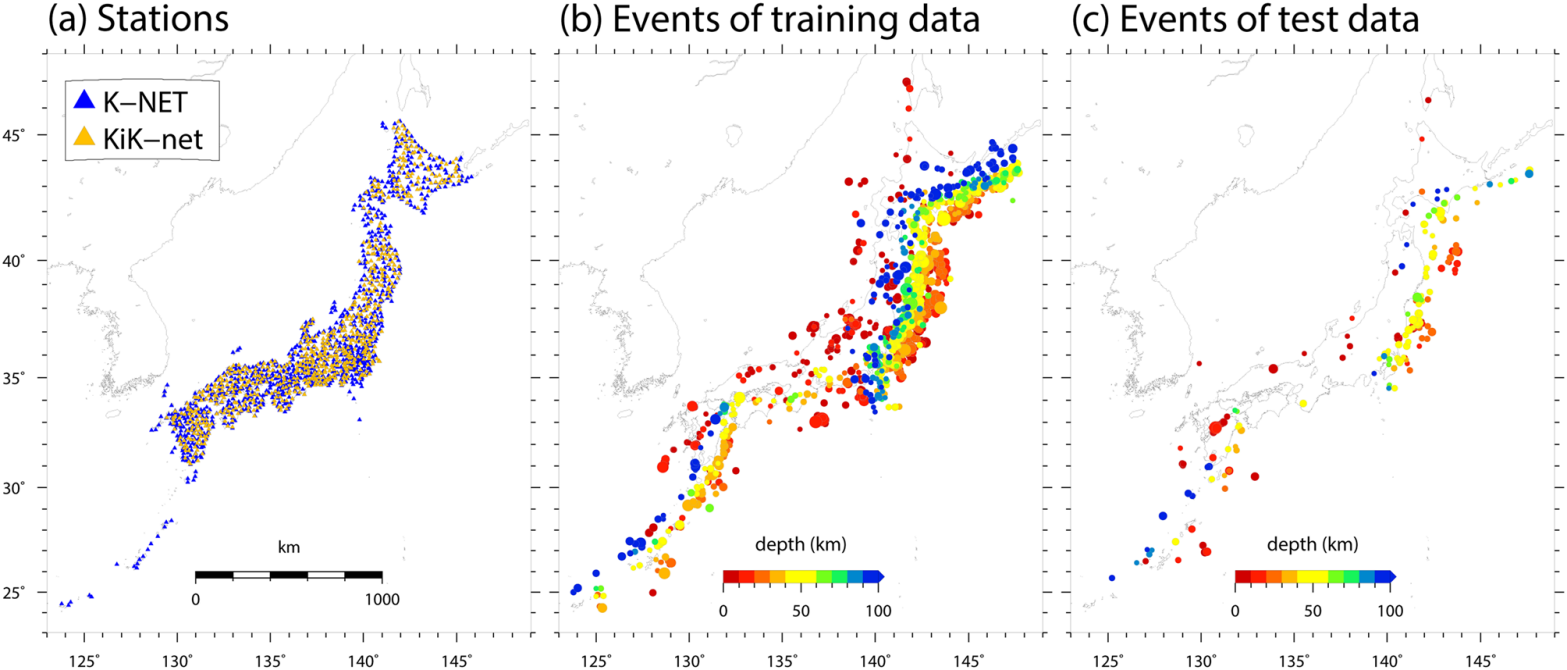
(**a**) Station distribution. Blue and yellow triangles indicate K-NET and KiK-net stations, respectively. (**b**) Spatial distribution of earthquake events in the training data. The circle color indicates the event depth. (**c**) Same as (**b**) but with test data.

The dataset used in this study is constructed by integrating open data in Japan provided by National Research Institute for Earth Science and Disaster Resilience (NIED): ground-motion records observed by K-NET and KiK-net^17,18^, earthquake source information provided by F-net^19,20^, and site information from Japan Seismic Hazard Information Station (J-SHIS)^21–24^. The dataset is divided into training data, with 186,310 samples (2082 events) recorded from 1997 to 2015, and test data, with 22,323 samples (208 events) recorded from 2016 to 2017. We design the test such that ground motions of future earthquakes are predicted by a predictor that has learned from records of past earthquakes. The volume of data used in this study is greater than in previous studies of GMPEs^7–11^. The station distribution of K-NET and KiK-net and the event distribution of the dataset are shown in Fig. 1.

PGA prediction is treated as a regression problem and the predictor is constructed using Extremely Randomized Trees (ERT)^25^. ERT is a tree-based ensemble ML method derived from Random Forest^26^, which fits multiple decision trees on various subsamples of data and combines them in determining the output to improve the predictive accuracy and control overfitting. ERT differs from Random Forest in the tree split approach: ERT randomly picks a node split (the variable index and variable splitting value are chosen randomly), whereas Random Forest finds the best split among a random subset of variables. ERT can decrease variance (the variability of model prediction for a given data) at the cost of a small increase in bias (the difference between the average prediction and the correct value)^25^.

**Figure 2:**
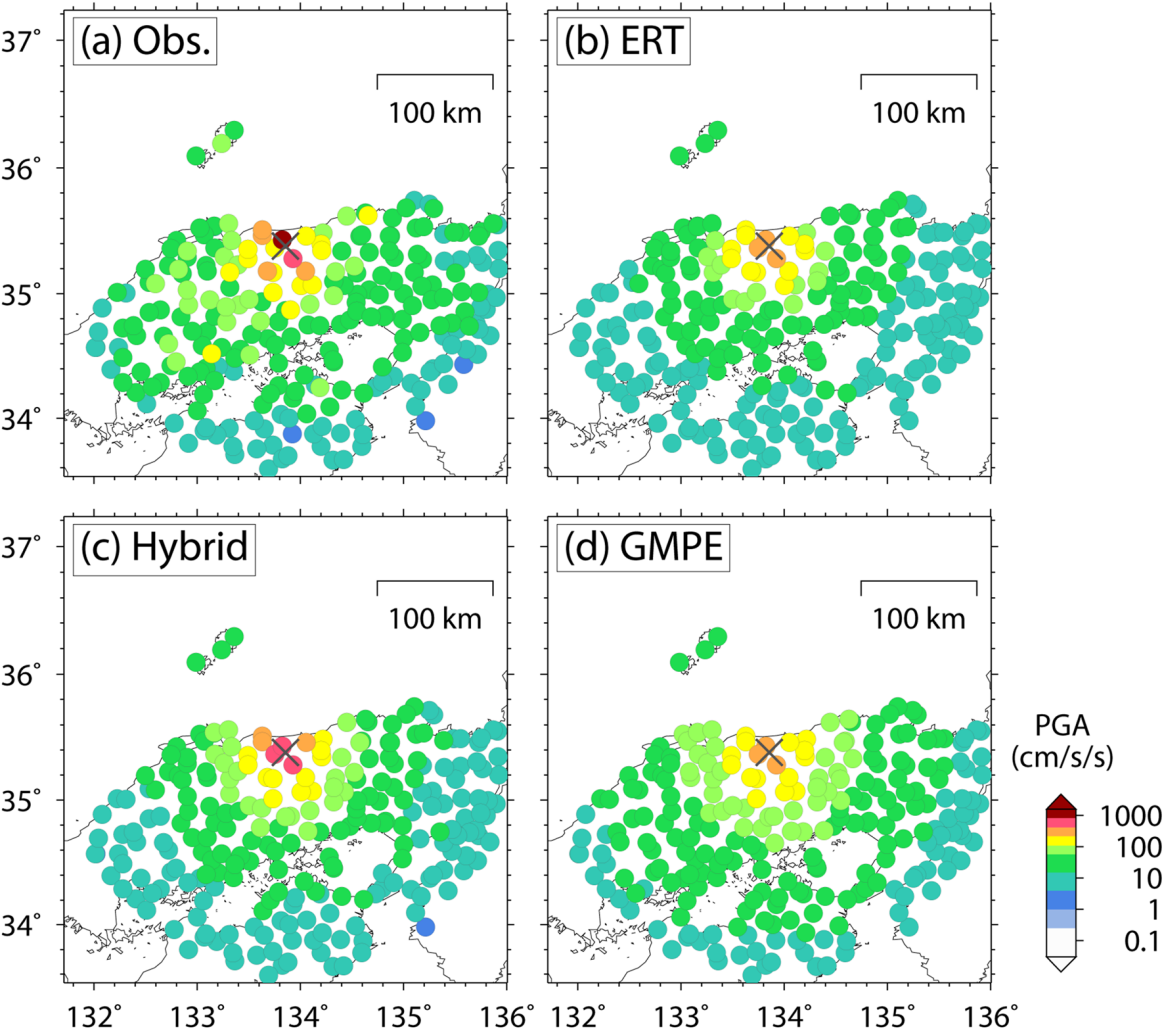
Spatial distribution of PGA for the 2016 central Tottori earthquake in (**a**) observation and prediction by (**b**) Extremely Randomized Trees (ERT) predictor, (**c**) hybrid predictor, and (**d**) GMPE. The cross indicates the epicenter of this event.

**Figure 3:**
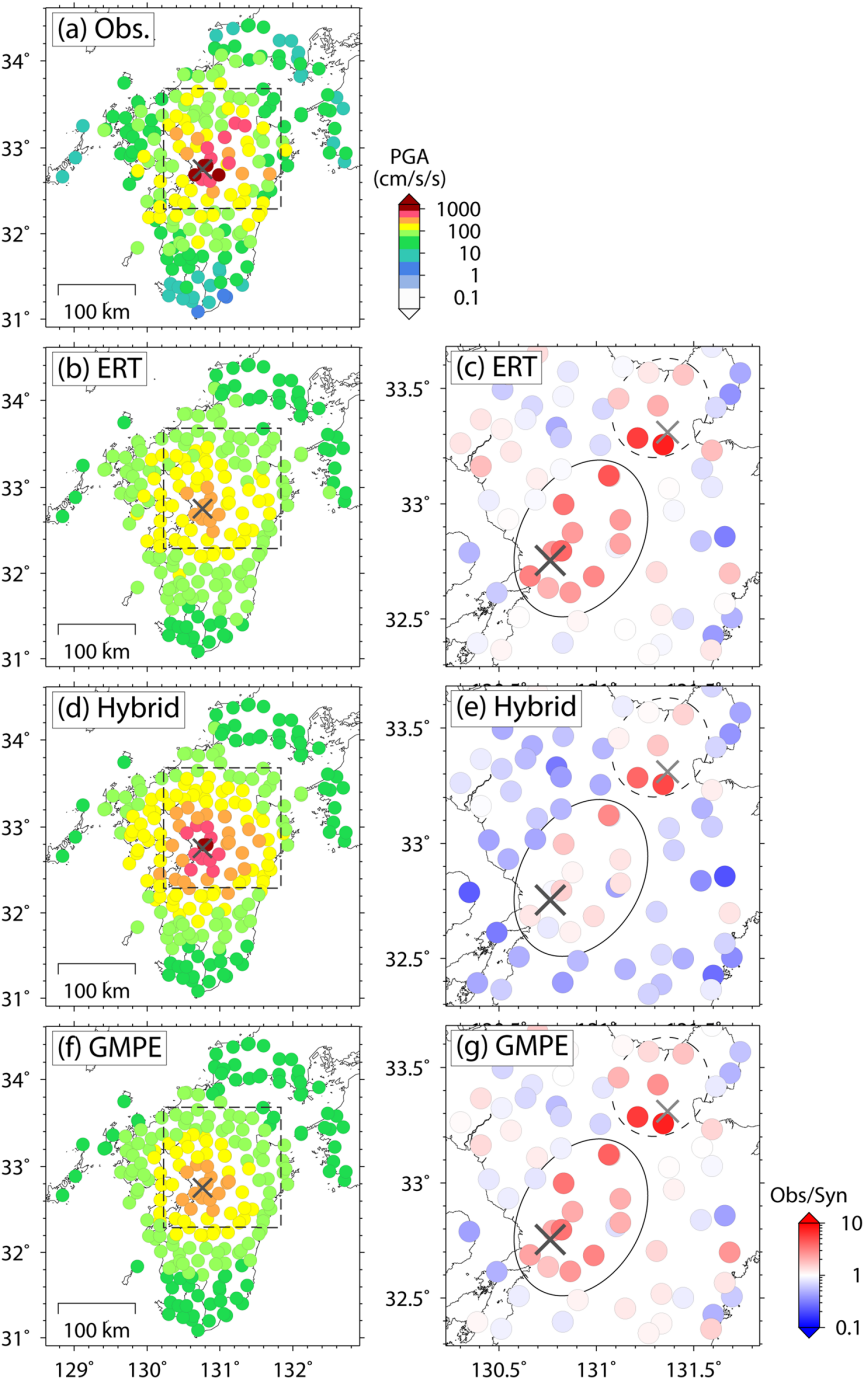
Spatial distribution of PGA for the 2016 Kumamoto earthquake in (**a**) observation and prediction by (**b**) ERT predictor, (**d**) hybrid predictor, and (**f**) GMPE, and the ratio distribution of observation and prediction by (**c**) ERT predictor, (**e**) hybrid predictor, and (**g**) GMPE. The figures on the right show an enlarged view of the dotted rectangular area in the left figures. The black cross indicates the epicenter of this event. The gray cross indicates the epicenter of the induced earthquake during this event, as determined by Suzuki et al.^29^.

The observations and predictions for earthquakes in the test data are compared to demonstrate the prediction performance of the obtained ERT predictor. Figure 2 shows the case of the 2016 central Tottori earthquake (*M*_w_ 6.2), a shallow crustal earthquake that caused strong motions with a maximum PGA of greater than 1,000 cm/s/s^27^. The overall feature of the observation is reproduced by the ERT predictor, although large PGA values of over 500 cm/s/s are not predicted. As another example, Fig. 3 shows the case of the 2016 Kumamoto earthquake (*M*_w_ 7.1), a shallow crustal earthquake that caused strong motions throughout Kyushu Islands, Japan, with a maximum PGA of greater than 1,000 cm/s/s and caused destructive damage to Kyusyu society by strong motions, surface ruptures, and subsequent landslides^28,29^. Figure 3 also shows the ratio distribution between observed and predicted PGAs. The predicted PGA distribution less than 200 cm/s/s is approximately consistent with the observations. However, the ERT predictor underestimates the observed strong motions around the epicenter (ellipse in Fig. 3c). Although the observed PGA values at several stations are close to or greater than 1,000 cm/s/s, the maximum PGA of the ERT predictor is no more than 500 cm/s/s. We identify another underestimation area denoted by a dotted ellipse in Fig. 3c; however, these strong motions were caused by a triggered earthquake that occurred just after the mainshock^29,30^ and are outside of the scope of this ground-motion prediction study.

**Figure 4:**
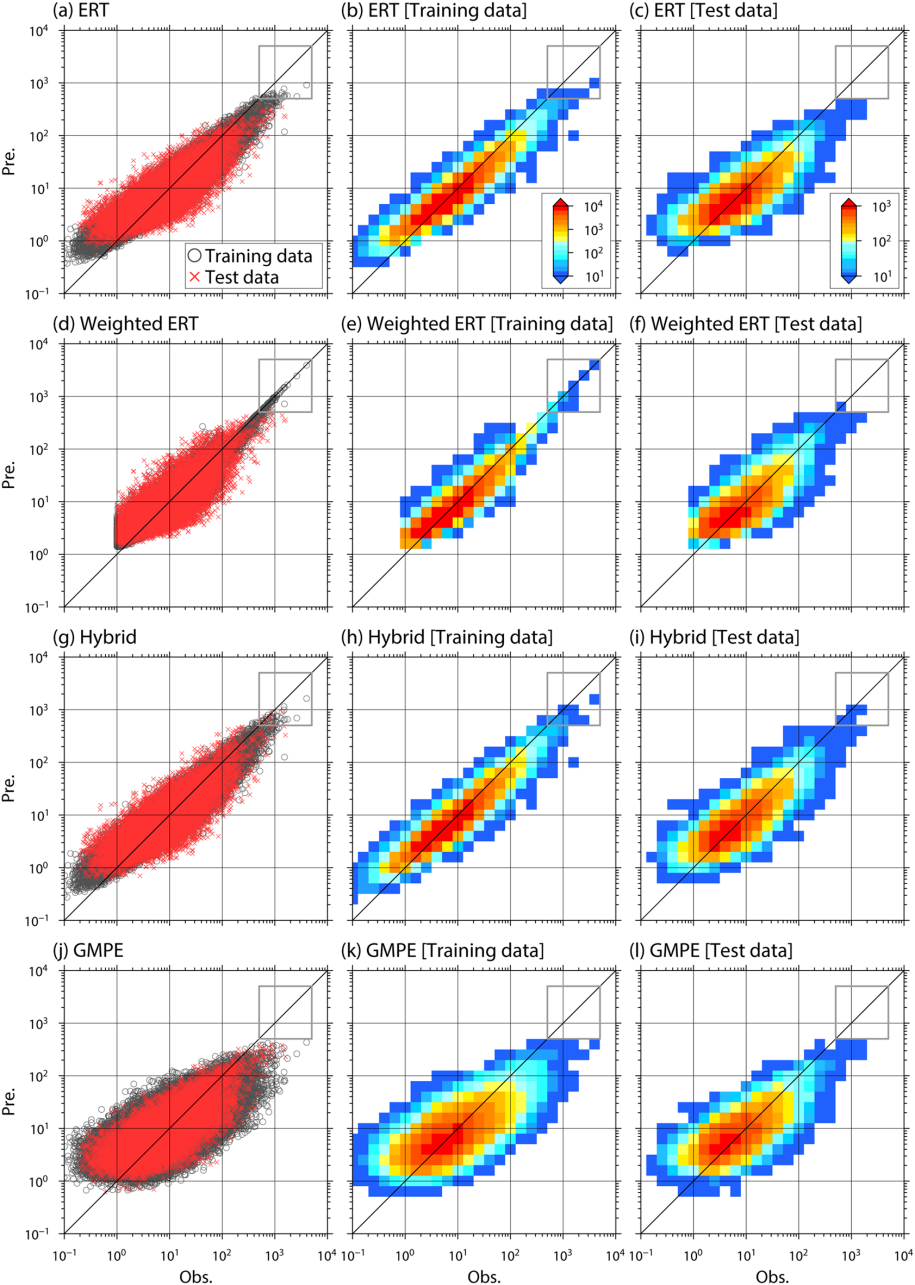
Relationship between observation and prediction in the training and test data (left), and heat maps showing the relationship in training data (central) and test data (right). The prediction method in each figure is (**a**–**c**) ERT predictor, (**d**–**f**) ERT predictor with weighted training data, (**g**–**i**) hybrid predictor, and (**j**–**l**) GMPE. Black circles and red crosses in the left-row figures indicate the training and test data, respectively.

**Figure 5:**
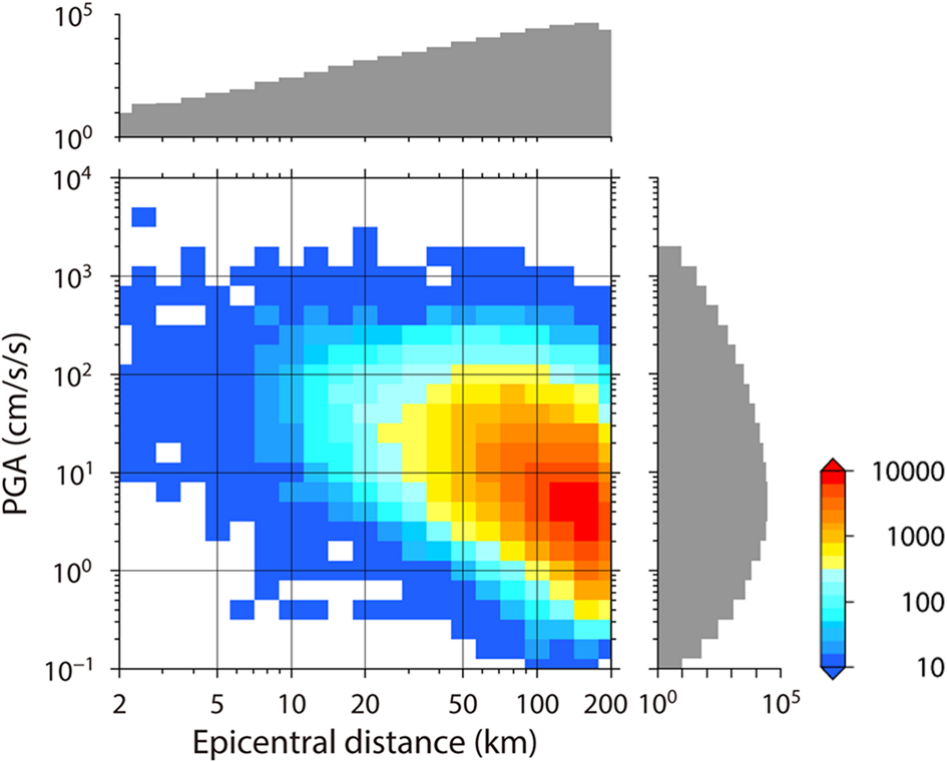
Heat map showing the relationship between epicentral distance and PGA in the training data with their histograms.

We also check the prediction performance on the whole dataset. Figure 4a–c show the relationship between observation and prediction by the ERT predictor. If there were no trends of underestimation and overestimation, the relationship would be distributed mainly on the diagonal line. As shown in the test data, however, the ERT predictor underestimates observed ground motions greater than 500 cm/s/s (gray square in Fig. 4c). This underestimation problem is also found in the training data (gray square in Fig. 4b). The cause of this underestimation problem is described as follows. As indicated in Fig. 5, which shows the distribution of the training data, the dataset used in this study is highly imbalanced. Ground-motion records in the range of 1–20 cm/s/s account for much of the dataset, while there are some records of strong motions (~ 1,000 cm/s/s). When the ML algorithm learns the imbalanced data, the learning focus is mainly on the fit of relatively weak ground motions. It diminishes the emphasis on the fit of strong motions. The underestimation problem for strong motions leads to a fatal flaw in the algorithm because strong motions generated by large earthquakes cause catastrophic damage to people and buildings, and the overlooking of such strong motions is unacceptable from an earthquake disaster resilience standpoint.

One approach to solving this problem involves weighting training data. This practice has been adopted in some previous studies of GMPEs^7,8^. We construct an ERT predictor that learns the weighted data based on the distribution of target PGAs. Figure 4d–f show one example of an ERT predictor learning weighted training data. Although this approach resolves the underestimation problem in the training data (Fig. 4e) and improves the data fit in the training and test data (Table 1), the underestimation trend remains in the test data (Fig. 4f). We confirm that the underestimation problem appears in other data-weighting cases. This can be explained as follows. Setting a large weight on specific records in training data is the same as replicating specific records. If an ML algorithm learns weighted data, this information is transferred to the ML predictor, which overfits multiple instances of an identical situation. Hence, the underestimation problem for strong motions in an ML-based predictor cannot be solved by data weighting. The relationship between distance and PGA is important for predicting ground-motion intensity, as indicated by the negative correlation in Fig. 5. However, the ERT predictor with weighted training data is unable to learn the relationship satisfactorily. We also confirm that ERT predictors with training data weighted based on epicentral distance retain the underestimation problem for strong motions. These results indicate that simply weighting data may not be helpful in ML, at least for the prediction of ground motions.

**Table 1:** Coefficient of determination (R^2^), total standard deviation (σ), between-events standard deviation (τ), and within-event standard deviation (φ) for each predictor.

Dataset	Predictor	R^2^	σ	τ	Φ
Training data	ERT	0.870	0.188	0.0812	0.169
	Weighted ERT^a^	0.913	0.173	0.0687	0.159
	Hybrid	0.872	0.187	0.0831	0.167
	GMPE	0.448	0.387	0.211	0.325
Test data	ERT	0.619	0.327	0.159	0.286
	Weighted ERT^b^	0.711	0.304	0.147	0.266
	Hybrid	0.643	0.317	0.167	0.270
	GMPE	0.465	0.388	0.208	0.329
Mean in the cross-validation test	ERT	0.590	–	–	–
	Hybrid	0.619	–	–	–

^a^The nonweighted training data without records of which the PGA is below 1 cm/s/s are used.

^b^The test data without records of which the PGA is below 1 cm/s/s are used.

### Hybrid predictor of machine learning and conventional ground motion prediction equation

To overcome the underestimation problem for strong motions, we propose a hybrid approach integrating ML and conventional GMPE. The advantage of conventional GMPEs is that they are stable for extrapolation or low data-density part because the regression equations are assumed based on the geophysical background of ground motions. However, these GMPEs are inflexible because the ground-motion model is strongly constrained by the preassumed function shape of the regression equations. On the other hand, nonparametric ML methods, such as Random Forest or ERT, are highly flexible despite having unreliable and unprovable prediction capability for extrapolation or low data-density parts. To retain the advantages of both approaches, we develop a hybrid predictor with basic prediction by a conventional GMPE followed by prediction using ERT.

Figure 4g–i suggest that this hybrid approach reduces the underestimation trend of observed strong motions in both training and test data. The underestimation trend around the epicenter of the 2016 Kumamoto earthquake is also improved (Fig. 3d,e). Moreover, the data fit of the hybrid predictor, shown in Table 1, is improved compared with the ERT predictor.

**Figure 6:**
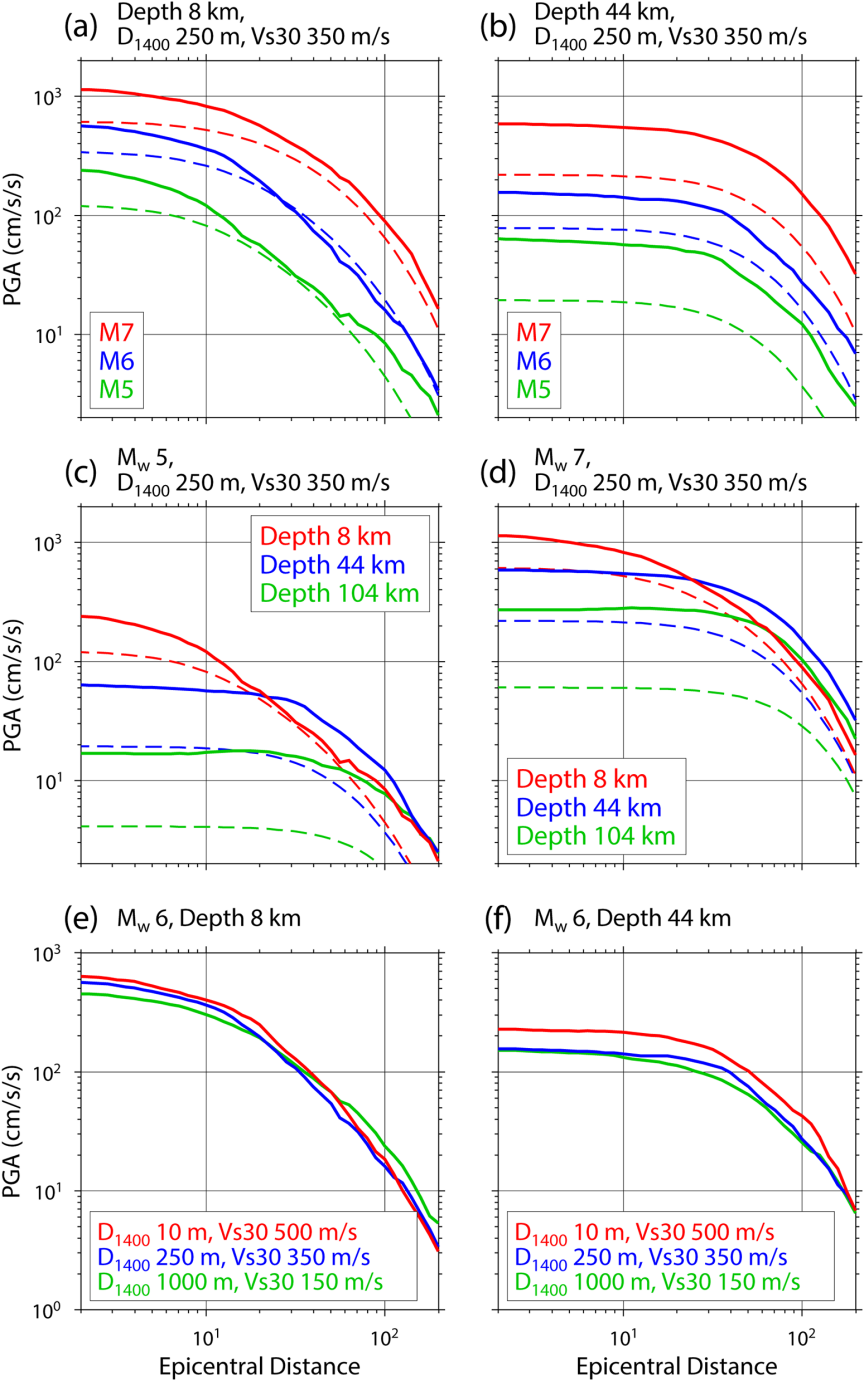
PGA prediction of the hybrid predictor as a function of epicentral distance. Broken lines indicate the prediction by the base model of Morikawa and Fujiwara^8^. The source and site information illustrated here are shown at the top of each figure.

**Figure 7:**
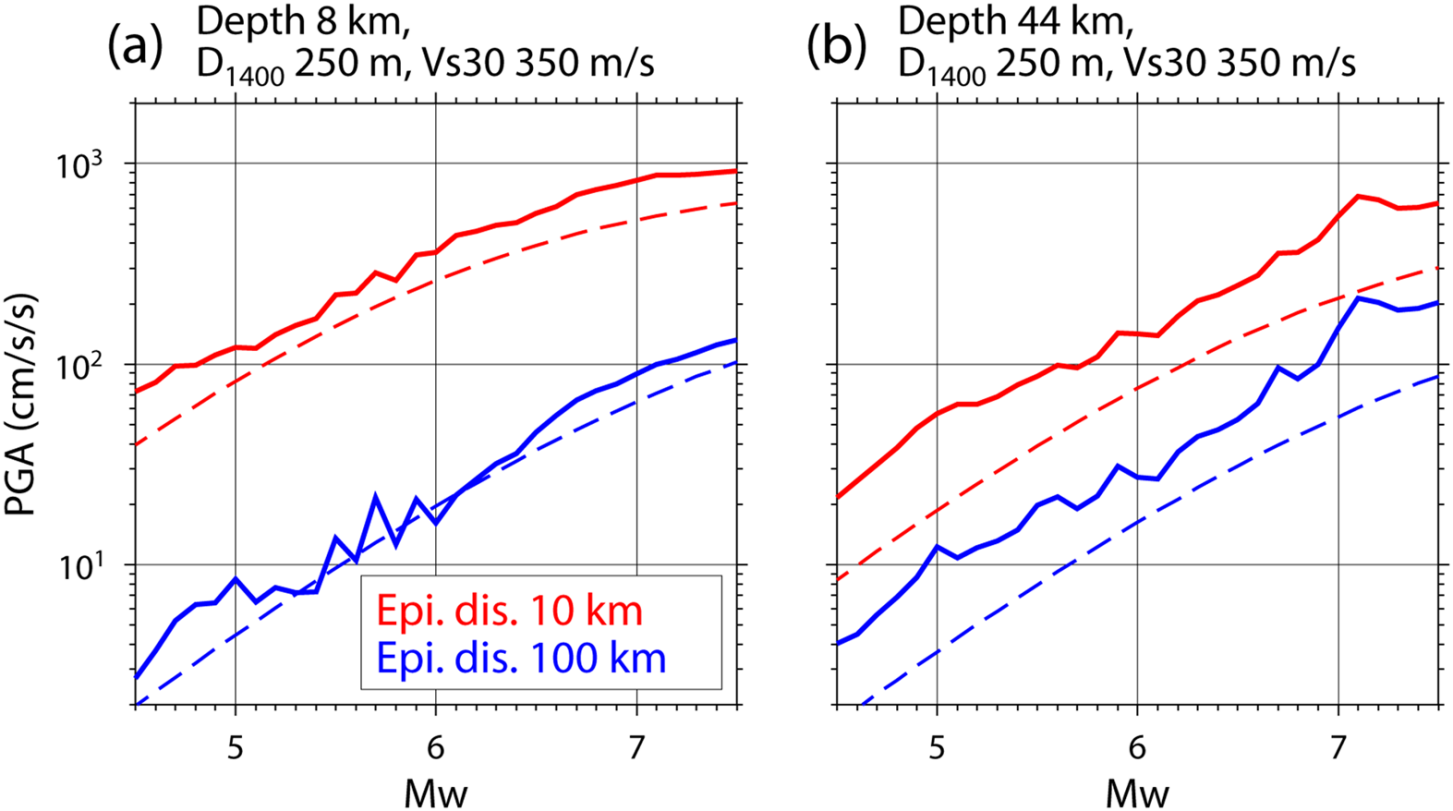
PGA prediction of the hybrid predictor as a function of *M*_w_. Broken lines indicate the prediction by the base model of Morikawa and Fujiwara^8^. The source and site information illustrated here are shown at the top of each figure.

Figures 6 and 7 show the PGA prediction of the hybrid predictor as a function of epicentral distance and *M*_w_, respectively. The distance attenuation and the magnitude scaling of PGA, which have been pointed out by many previous studies for ground motions, hold up in the hybrid predictor. Moreover, the relationships seem to depend on event depth. The PGA for deep events is large compared to that of shallow events at large epicentral distances (Fig. 6c,d). This dependence of ground-motion intensity on event depth may be explained by the depth dependence of the stress drop^31,32^ or the depth dependence of the nongeometric attenuation of seismic waves in the lithosphere^33,34^ or both.

In Fig. 6e,f, the prediction curves for sites of (*D*_1400_, *Vs30*) = (10 m, 500 m/s), (250 m, 350 m/s), and (1,000 m, 150 m/s) are shown. This comparison indicates that the predicted PGA values at hard sites with a thin sedimentary layer (large *D*_1400_) and hard near-surface ground conditions (small *Vs30*) are larger than those at soft sites with a thick sedimentary layer and soft near-surface ground conditions. This can be explained as follows: The PGA is determined mainly by the high-frequency components of seismic waves, and the high-frequency seismic waves tend to be amplified at a thin sediment layer based on the quarter-wavelength rule^35,36^. The site effect predicted by this study contradicts previous studies^8,15^ that the ground-motion intensity at hard sites tends to be smaller than at soft sites. These studies explained the site amplification effect by a single or several independent site-condition parameters, while the site amplification effect in this study is represented by the combination of the two site-condition parameters (*D*_1400_, *Vs30*) that interact with each other. Further studies about how to represent the site amplification effect in the ML model are required. The hybrid predictor also suggests that the variation of relative site amplification for an earthquake at a depth of 44 km is larger than that for an earthquake at a depth of 8 km, which implies the dependence of site amplification on the event depth. This dependence, which may be caused by the event-depth dependence of the incident angle of a seismic ray to the Earth’s surface, was not considered in previous GMPEs.

## Discussion

We constructed a GMPE based on the conventional regression analysis^8^ from the training data. The prediction results are shown in Table 1 and Figs. 2, 3, and 4. The explanatory variables of this GMPE are the same as those of the ML-based predictors proposed in this study. The prediction performance of the GMPE for the training and test data is not as good as that for the ML-based predictors (Table 2). Furthermore, an underestimation problem for strong motions is found (Figs. 4j–l). Prediction examples for real earthquakes, shown in Figs. 2 and 3, indicate that although the overall trend is reproduced, the GMPE prediction for the 2016 Kumamoto earthquake underestimates strong motions. Although it would be premature to conclude which method is superior, the results of this study suggest that an ML predictor has the potential to surpass a conventional GMPE.

**Table 2:** Parameter setting of extra trees regressor.

Number of trees	Maximum tree depth	Minimum number of samples required to be at a leaf node	Number of features to consider when looking for the best split
1,000	50	2	2

The data fit of the ML-based predictors is worse in the test data than in the training data (Fig. 4 and Table 1). The same trend occurs even when a different setting is used for the parameters in ERT. This implies that although their variances are not large, the obtained ML-based predictors have an overfitting trend. Even though we reduced the variance by the introduction of ERT, the overfitting trend remains. One likely reason is that records from the two-decade-long ground-motion observations in K-NET and KiK-net used in this study may be insufficient to capture all patterns of earthquakes and ground motions because of the spatial and temporal high heterogeneity of seismic activity. To fundamentally solve this problem, an augmentation of data volume is needed. This study used only K-NET and KiK-net data; therefore, it is important to assemble ground-motion records from other institutes in Japan and construct a ground-motion database for all of Japan, such as the NGA-West2 database^37^. It is also necessary to continue accumulating ground-motion records and develop a ground-motion dataset where simulation-based data are integrated with observation-based data. Another likely reason for the overfitting trend is that the five explanatory variables (*D*, *M*_w_, *H*, *D*_1400_, *Vs30*) are not sufficient to fully explain the PGA, although they can reproduce the overall PGA trend. The real generation mechanism of ground motions is more complex than the assumed model in this study, where ground motions depend on only the five variables. The uncertainty of each variable also affects the ML model. Further investigation about these points is needed and ML technologies can contribute to it. Although the overfitting trend of the ML predictors is the focus of this paragraph, we consider that the overfitting trend is not a large problem from the perspective of the prediction problem for ground-motion intensity. The prediction examples of the hybrid predictor seem to be reasonable and stable as demonstrated in Figs. 6 and 7. Additionally, the data fitting for the test data in the hybrid predictor is better than that in the GMPE (Table 1) and the variance in the ML predictors is not large.

This study demonstrated that the hybrid approach improves the underestimation trend for strong motions, which is caused by the bias in the ground-motion dataset. However, the underestimation problem is still not solved completely. Moreover, the ground-motion data have other biases including the imbalanced distribution of the epicentral distance and *M*_w_. Augmentation of data volume, especially strong ground motions, and the attempts to investigate how to handle the imbalanced dataset in the regression problem are needed to approach these problems.

Kong et al.^1^ claimed that the integration of ML and physical models produces a synergy that balances the complementary strengths of physical intuition with data-driven insights. This study demonstrated that the hybrid approach of ML and the conventional GMPE improves the underestimation problem for strong motions and leads to better prediction performance than a predictor using only ML or the conventional GMPE. We consider that the hybrid approach of ML and physical models is also useful for predictions in other fields, particularly where biased datasets are being used. Moreover, the output from this hybrid approach suggests new insights into the relationship between ground-motion intensity and site condition, as ML can express the complex relationship among explanatory and response variables without prior information. The application of ML has the potential to enhance data-driven discovery^2^.

## Methods

### Dataset

For PGA data, we referred to records of the national strong motion network in Japan, deployed and operated by NIED, K-NET and KiK-net^17,18^. The ground-motion records have been publicly available on the associated website since May 1996 for K-NET and October 1997 for KiK-net. For earthquake source information (event location and moment magnitude), we referred to the F-net moment tensor solution catalog^19,20^, which has also been publicly available on the associated website since January 1997. For event depth, we used the centroid depth of F-net. In this study, an earthquake is regarded as a point source, and we ignored the source finiteness of earthquakes to simplify the prediction problem. For *D*_1400_ and *Vs30* information at each site, we referred to the site-below underground information from a deep subsurface structure model of Japan^22^ and the *Vs30* map of Japan suggested by Matsuoka and Wakamatsu^23^, both of which are publicly available on the website of J-SHIS^21,24^.

To construct the dataset, we first collected available ground-motion data observed by K-NET and KiK-net and event data from the F-net moment tensor solution catalog and unified hypocenter catalog provided by the Japan Meteorological Agency (JMA). Then, we integrated these data and added site information (*D*_1400_ and *Vs30*) for each station. Finally, we retrieved ground-motion records for events satisfying the following conditions: (1) 4.5 ≤ *M*_w_ ≤ 7.5, (2) epicentral distance shorter than 200 km, (3) event depth shallower than 200 km, and (4) ground-motion records observed from at least five stations. At this step, we eliminated ground-motion records that did not include the S-wave part by checking the theoretical arrival time of the S-wave. The upper limit of *M*_w_ was set because the effect of the source finiteness in huge earthquakes (*M* 8 or 9) is expected to be large and the assumption of point source does not hold in very large earthquakes. The upper limit of the event depth was set to exclude deep earthquakes, which cause anomalous distributions of ground-motion intensity^38,39^, from the dataset. The upper limit of epicentral distance was the same as in Morikawa and Fujiwara^8^. We separated the dataset into training data with 186,310 samples (2082 events) consisting of records from 1997 to 2015 and test data with 22,323 samples (208 events) consisting of records from 2016 to 2017. For data preprocessing, we took the common logarithm of the epicentral distance, event depth, and PGA and then standardized the five explanatory variables and the target variable.

### ERT predictor

In constructing the ERT predictor, we used the Extra Trees Regressor in the scikit-learn Python programming package^40^. The parameter setting of Extra Trees Regressor is the same as that used in the hybrid predictor, which is mentioned later.

### ERT predictor with weighted training data

For data weighting, the training data were divided into four groups; G1: 1 cm/s/s ≤ PGA < 10 cm/s/s, G2: 10 cm/s/s ≤ PGA < 100 cm/s/s, G3: 100 cm/s/s ≤ PGA < 1,000 cm/s/s, and G4: PGA ≥ 1,000 cm/s/s. G1–G4 had 113,806, 60,698, 3,891, and 22 records, respectively, in the training data (13,581, 7,243, 518, and 3 records, respectively, in the test data). We excluded records where the PGA was below 1 cm/s/s. From the data groups, a weighted dataset was prepared where the records of each group were replicated depending on their group weights. For example, a group weight of 2 means duplicating the group records. In this data weighting, a data group with a small volume of data was weighted heavily. Then, the weighted dataset was learned by the ML algorithm. This cycle was repeated using different weight combinations on the dataset and checking the ML results. Figure 4 provides an example with weighted training data where the weights of G1, G2, G3, and G4 were one, one, four, and sixteen, respectively.

### Hybrid predictor of ERT and GMPE

We developed a hybrid predictor where the predicted PGA is represented by adding the predicted value of an ML predictor to the predicted value of a conventional GMPE. For the GMPE, we used base model 1 for crustal earthquakes in Morikawa and Fujiwara^8^. Their variables are the hypocentral distance $\left( {\sqrt {D^{2} + H^{2} } } \right)$ and *M*_w_. The ML predictor with the variables *D*, *H*, *M*_w_, *D*_1400_, and *Vs30* learned the residual between the observation and GMPE prediction as the training data. For the ML algorithm, we used the Extra Trees Regressor in the scikit-learn Python programming package^40^. Table 2 indicates the corresponding parameter setting. The number of trees was set to 1,000 because of the performance limit of the machine server we used. The maximum depth of the tree, the minimum number of samples required to be at a leaf node, and the number of features to consider when looking for the best split were determined based on the balance of the data fit and variance in the cross-validation test. In the cross-validation test, because seismic records are strongly correlated in time series and the use of random data split may cause results to be misunderstood, we sequentially split the training data into 10 groups based on the time series of events and conducted K-fold cross-validation test using 10 data groups.

### GMPE

Following Morikawa and Fujiwara^8^, we reconstructed the GMPE considering site amplification due to deep sedimentary layers and shallow soft soils:

1 $$\log PGA = G_{o} + G_{d} + G_{s}$$

2 $$G_{o} = a \cdot \left[ {{\min}\left( {M_{w} ,M_{0} } \right) - M^{\prime } } \right]^{2} + b \cdot X + c - \log \left[ {X + d \cdot 10^{{e \cdot {\min}\left( {M_{w} ,M_{0} } \right)}} } \right]$$

3 $$G_{d} = p_{d} \cdot \log \left[ {{\max}\left( {D_{1400 \, min} ,D_{1400} } \right)/D_{0} } \right]$$

4 $$G_{s} = p_{s} \cdot \log \left[ {{\min}\left( {V_{s \,max} ,V_{s} 30} \right)/V_{0} } \right]$$

where $G_{o}$ is the base GMPE model with $M_{w}$ and hypocentral distance $X \left( { = \sqrt {D^{2} + H^{2} } } \right)$, $G_{d}$ is a correction term for site amplification by deep sedimentary layers with *D*_1400_, $G_{s}$ is a correction term for site amplification by shallow soft soils with *Vs30*, and $\left( {a,b,c,d,e,M_{0} ,M^{\prime } ,p_{d} ,D_{1400 \,min} , D_{0} ,p_{s} ,V_{s \,max} ,V_{0} } \right)$ are parameters to be determined. In these parameters, $\left( {d,e,M_{0} ,M^{\prime } , D_{0} ,V_{0} } \right)$ is same as in Morikawa and Fujiwara^8^. The rest of the parameters are determined by the following scheme. First, $\left( {a, b, c} \right)$ in Eq. (2) is determined by the least-squares method from the training data with a distance-based weighting scheme8. Then, $\left( {p_{d} ,D_{1400 \,min} } \right)$ in Eq. (3) is determined by the least-squares method and grid search of the residuals between the observations and $G_{o}$ in the training data. Finally, $\left( {p_{s} ,V_{s \,max} } \right)$ in Eq. (4) is determined by the least-squares method and grid search of the residuals between the observations and $G_{o} + G_{d}$ in the training data.

### Measurement of predictor performance

To measure the predictor performance, we used the coefficient of determination $R^{2}$:

5 $$R^{2} = 1 - \frac{{\mathop \sum \nolimits_{i = 1}^{n} \mathop \sum \nolimits_{j = 1}^{{m_{i} }} \left( {o_{ij} - p_{ij} } \right)^{2} }}{{\mathop \sum \nolimits_{i = 1}^{n} \mathop \sum \nolimits_{j = 1}^{{m_{i} }} \left( {o_{ij} - \overline{o}} \right)^{2} }}$$

where *n* is the number of earthquakes, $m_{i}$ is the number of recordings for the *i*th earthquake, $o_{ij}$ is the observed value of $\log_{10} PGA$ for the *i*th earthquake at the *j*th site, $p_{ij}$ is the predicted value, and $\overline{o}$ is the mean of the observed data. We also calculated the total standard deviation ${\upsigma }$, the between-event standard deviation $\tau$, and the within-event standard deviation $\phi$. The total error of the GMPE is decomposed into the between-event and within-event errors, which are zero-mean, independent, normally distributed random variables with standard deviations $\tau$ and $\phi$, respectively^41,42^. The between- and within-events residuals are assumed uncorrelated, so σ can be written as:

6 $${\upsigma } = \sqrt {\tau^{2} + \phi^{2} } .$$

To estimate values of the standard deviations, first, we calculated the residuals $r_{ij}$:

7 $$r_{ij} = o_{ij} - p_{ij} .$$

From $r_{ij}$ for all data, the total standard deviation ${\upsigma }$ was obtained. The between-event error for each earthquake can be described as follows^42,43^:

8 $$\eta_{i} = \frac{{\tau^{2} \mathop \sum \nolimits_{j = 1}^{{m_{i} }} r_{ij} }}{{m_{i} \tau^{2} + \phi^{2} }}$$

where $\eta_{i}$ is the between-event error for the *i*th earthquake. This equation implies that if there are a large number of recordings from an earthquake, the between-event error can be approximated by the mean residuals for that event:

9 $$\eta_{i} \approx \frac{{\mathop \sum \nolimits_{j = 1}^{{m_{i} }} r_{ij} }}{{m_{i} }}.$$

Using Eq. (9), we obtained $\eta_{i}$ for earthquakes for which $m_{i}$ is larger than 100. Then, from $\eta_{i}$ for the selected earthquakes, we estimated the between-event standard deviation $\tau$ and obtained the within-event standard deviation $\phi$ using Eq. (6).

## Data Availability

Ground motion records at K-NET and KiK-net^18^ are available at https://www.kyoshin.bosai.go.jp/. The moment tensor solution catalog of F-net^20^ is available at https://www.fnet.bosai.go.jp/. The subsurface structure model for Japan is available from J-SHIS^24^ at https://www.j-shis.bosai.go.jp/.
